# Cellular communication through extracellular vesicles and lipid droplets

**DOI:** 10.1002/jex2.77

**Published:** 2023-03-01

**Authors:** Irumi Amarasinghe, William Phillips, Andrew F. Hill, Lesley Cheng, Karla J. Helbig, Eduard Willms, Ebony A. Monson

**Affiliations:** ^1^ School of Agriculture, Biomedicine and Environment La Trobe University Melbourne Australia; ^2^ La Trobe Institute for Molecular Sciences La Trobe University Melbourne Australia; ^3^ Institute for Health and Sport Victoria University Footscray Victoria Australia

**Keywords:** cellular communication, extracellular vesicles, lipid droplets

## Abstract

Cellular communication is essential for effective coordination of biological processes. One major form of intercellular communication occurs via the release of extracellular vesicles (EVs). These vesicles mediate intercellular communication through the transfer of their cargo and are actively explored for their role in various diseases and their potential therapeutic and diagnostic applications. Conversely, lipid droplets (LDs) are vesicles that transfer cargo within cells. Lipid droplets play roles in various diseases and evidence for their ability to transfer cargo between cells is emerging. To date, there has been little interdisciplinary research looking at the similarities and interactions between these two classes of small lipid vesicles. This review will compare the commonalities and differences between EVs and LDs including their biogenesis and secretion, isolation and characterisation methodologies, composition, and general heterogeneity and discuss challenges and opportunities in both fields.

## 1 INTRODUCTION

Communication between cells and organelles within cells through lipid particles is an integral physiological process. One mechanism cells use to communicate occurs through the release and uptake of extracellular vesicles (EVs). Over the last decade EVs have emerged as mediators of cellular communication and they are actively researched for their cargo transferring capabilities and as potential therapeutics and diagnostics in diseases such as cancer (Becker et al., 2016) and neurodegenerative disorders (Asai et al., 2015; Cervenakova et al., 2016; Prusiner et al., 2015). Small sized EVs, historically termed exosomes, are the most well studied population of EVs. Still, recent advancements in isolation and analysis techniques have resulted in the discovery of novel lipid vesicle populations and other extracellular particles (e.g., exomeres) with diverse traits (Zhang et al., 2018).

In addition to releasing EVs, cells contain numerous intracellular lipid vesicles which have similarities in their structure, size and overall composition EVs. Lipid droplets (LDs) (also known as lipid bodies, oil bodies or adiposomes) are small ubiquitous organelles which are essential to cellular lipid metabolism and energy homeostasis. LDs have also been demonstrated to play important roles in multiple biological processes including vesicular trafficking, protein folding, protein storage, autophagy, virus replication and more recently in immunity (as reviewed in Monson, Crosse, et al., 2021; Olzmann & Carvalho, 2019; Welte, 2007). There is evidence emerging that suggests LDs can be secreted into the extracellular space by cells, including LDs being secreted from milk duct cells into milk (Lu et al., 2022), as well as evidence of LDs transferring between epithelial cells in vitro (KB HeLa cells) (Collot et al., 2018). It was also recently hypothesised that LDs might be packaged within adipocyte‐derived EVs (termed AdExos by the authors), allowing them to activate macrophages in surrounding adipose tissue (Flaherty et al., 2019). Interestingly, multilamellar vesicles (i.e., vesicles with more than one membrane), likely to arise from vesicles being packed inside each other, have been observed with CryoTEM in samples of isolated EVs (Coleman et al., 2012; Emelyanov et al., 2020; Höög & Lötvall, 2015; Yuana et al., 2013; Zabeo et al., 2017). The mechanisms underlying the aforementioned vesicle structures and their biological roles remain to be determined.

Based on the similarities between EVs and LDs and the emerging role of LDs in intercellular communication, there is a clear synergy between these cellular particles. With the ongoing discovery of novel extracellular particles, there has been difficulty with the nomenclature surrounding the EVs and other cellular particles. Throughout this review we therefore use the term ‘lipid vesicles’ to collectively refer to both bilayer and monolayered vesicles, in particular: EVs and LDs. This review focuses on the similarities and differences between these two lipid vesicles in terms of their characteristics and methods used to investigate them, with the aim to support and encourage interdisciplinary research.

## 2 BIOGENESIS AND SECRETION OF LIPID VESICLES

**FIGURE 1:**
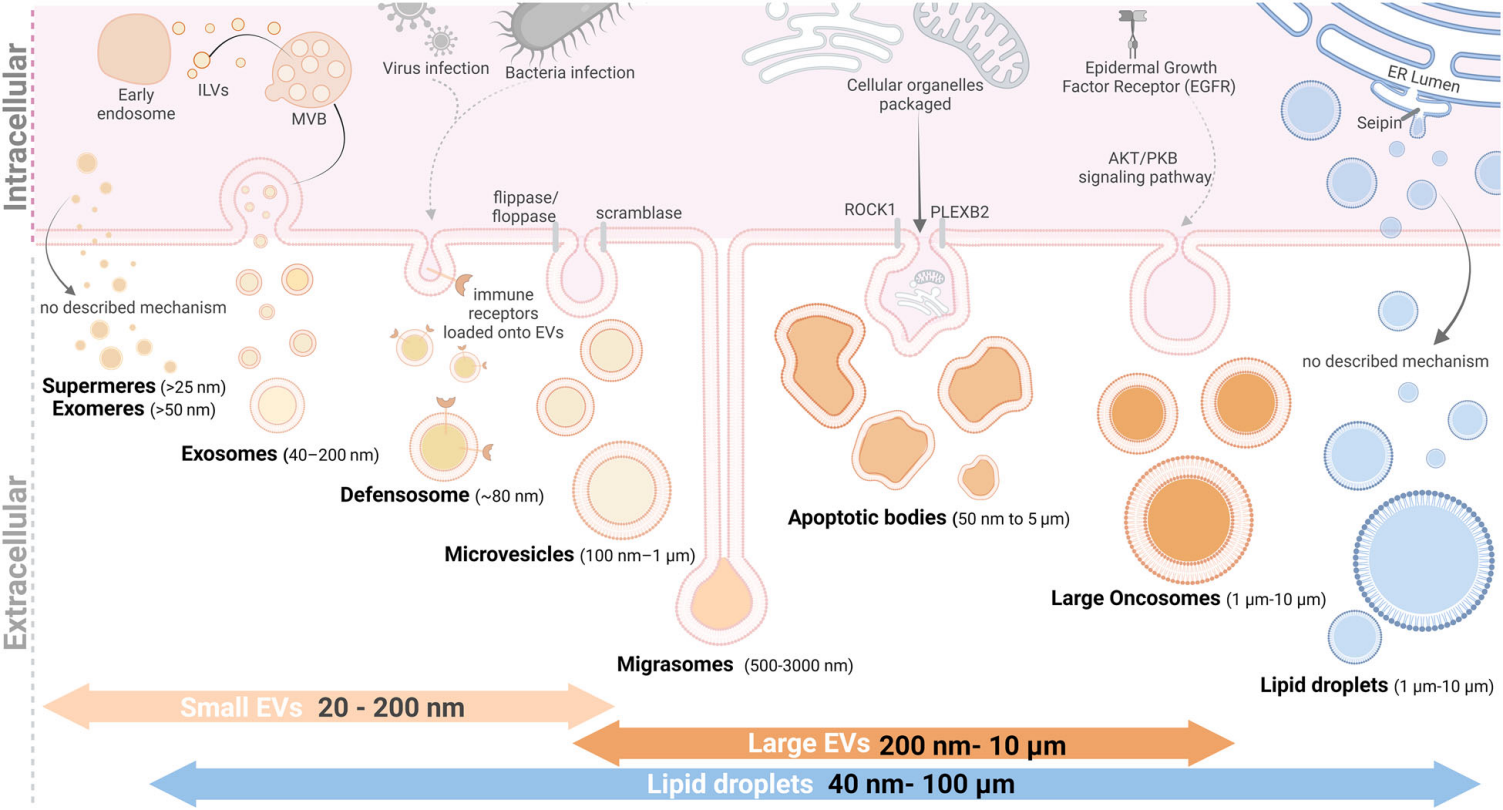
Formation and characteristics of the diverse types of lipid vesicles that play a role in cellular communication. Classifying EVs by their biogenesis is complex to achieve due to the lack of consensus on their unique markers and their overlap in size. Minimal information for studies of extracellular vesicles (MISEV) guidelines have suggested to refer to EV subtypes as either small or large EVs (Théry et al., 2018). According to the guidelines small EVs are between <20 nm or <200 nm and exomeres, supermeres, exosomes and defensosomes fall into this category. Large EVs are >200 nm which include microvesicles (100 nm–1 μm), migrasomes (500–3000 nm), apoptotic bodies (50 nm–5 μm), and large oncosomes (1–10 μm). LDs are heterogenous in size ranging from 1 to 10 μm. ER, endoplasmic reticulum; ILV, intraluminal vesicle; MVB, multivesicular body.

Lipid vesicles in general are highly heterogeneous. This heterogeneity can be underpinned by their biogenesis, which can influence their size, structure, the cargo they carry and subsequently the effect they have on target cells. This heterogeneity does not just exist between different groups of lipid vesicles (e.g., EVs, LDs) but also within lipid vesicles of the same group (e.g., there is size and cargo heterogeneity between exosomes). This has made it difficult to classify lipid vesicles into groups for characterisation and functional studies. During the last few decades, the interest in EVs and their applications has grown considerably, with there being a lot of focus on the functional role of EV heterogeneity, prompting discussion on the best way to group these lipid vesicles. For example, EVs can be grouped based on their size, but are also grouped based on their biogenesis and composition, with different subtypes of EVs having complex, and different mechanisms of biogenesis (EL Andaloussi et al., 2013) (Figure 1). In contrast, there is less known about LD heterogeneity. LDs are highly heterogeneous in size, and also in the cargo they carry (Figure 1). In general LDs have one well described mechanism of biogenesis, however, the activators of this biogenesis can differ and is thought to influence their protein cargo (Bosch et al., 2020; Liu et al., 2019)

EVs and LDs are both highly heterogenous, however there are similarities in their structure and size (Figure 1). Not much is known about the similarities between these two lipid vesicle groups, and as technologies to characterise these particles are becoming more sensitive, it is important to understand the overlap between these lipid vesicles.

### 2.1 Extracellular vesicles

Since the discovery of EVs, researchers have assigned various names for the particles they isolated and investigated. As a result, the nomenclature and classification of EVs has been an ongoing point of research and discussion (Gould & Raposo, 2013; Witwer & Théry, 2019). The International Society for Extracellular Vesicles (ISEV) was established with the aim of improving scientific rigour and has led to consensus on nomenclature and other aspects of EV research. On the basis of this, the society provides the field with a set of guidelines, The MISEV guidelines (The Minimal Information for Studies of Extracellular Vesicles), which is periodically updated and provides recommendations on aspects of EV research, including nomenclature (Théry et al., 2018; Witwer et al., 2021). As outlined in the document: ‘ISEV endorses “extracellular vesicle” (EV) as the generic term for particles naturally released from the cell that are delimited by a lipid bilayer and cannot replicate, i.e., do not contain a functional nucleus’.

As highlighted before, EVs are heterogeneous and the classification of EVs into distinct populations or subtypes is challenging. As is also outlined in the MISEV guidelines, specific markers that allow research to accurately differentiate between EV populations have not been established. As a result, it is in practice extremely difficult to accurately assign EVs to a distinct biogenesis pathway. Nevertheless, classification on this basis into endosome‐derived exosomes and plasma membrane‐derived microvesicles has been the predominant classification applied throughout the scientific literate. To address this discrepancy, it is therefore recommended to classify EVs according to their size, into small EVs (<200 nm) or large EVs (>200 nm), unless it is possible to establish specific markers of subcellular origin that are reliable within the experimental system(s) (Théry et al., 2018).

Multiple biogenesis pathways may underly the formation of EVs, up until now several pathways have been described (van Niel et al., 2018). EVs can originate from the endocytic pathway, where the formation of intraluminal vesicles inside multivesicular bodies (endosomal compartments) occurs via the invagination of the endosomal membrane (Pan & Johnstone, 1983). The formation of intraluminal vesicles is mainly driven by the ESCRT (Endosomal Sorting Complex Required for Transport) machinery, a cytoplasmic multi‐subunit system essential for intraluminal vesicle membrane remodelling, enabling budding and cargo sorting in multivesicular bodies. Intraluminal vesicles are then secreted upon fusion of the multivesicular bodies with the plasma membrane, releasing the EVs into the extracellular space (Johnstone et al., 1987). There are also several alternate mechanisms of EV biogenesis, including complex lipids such as ceramides which can alter the membrane structure of EVs contributing to budding to form intraluminal vesicles (Trajkovic et al., 2008), and other membrane proteins such as tetraspanins and Rab GTPases contributing to the trafficking and release of EVs from the plasma membrane (Simons & Raposo, 2009).

Formation of EVs directly from the plasma membrane can also occur, these EVs have historically been referred to as microvesicles. It has been shown that an increase in intracellular calcium (Ca^2+^) concentrations can trigger a change in the lipid distribution within the trans‐bilayer. This process is carried out via the three membrane bound lipid transporters: flippase, floppase and scramblase, which impede membrane‐cytoskeletal anchorage. This promotes regions on the membrane to form blebs, that are released as EVs into the extracellular environment once the vesicle neck undergoes scission, a process involving ESCRT proteins (Taylor & Bebawy, 2019).

Apoptosis can trigger a cell disassembly process which is at the basis of the biogenesis of apoptotic bodies. The formation of this EV population is initiated by membrane blebbing, that is, mediated by protein kinases such as Rho‐associated protein kinase (ROCK1) followed by protrusion formations, including the formation of apoptopodia and beaded apoptopodia. These unique protrusions of apoptotic cells lead to the formation of apoptotic bodies (Atkin‐Smith et al., 2015). Interestingly, this formation process can also result in the packaging of organelles such as the Golgi apparatus, endoplasmic reticulum and mitochondria into apoptotic bodies (Gregory & Dransfield, 2018; Jiang et al., 2017). This finding indicates the potential lipid vesicles end up being packaged into apoptotic bodies.

#### 2.1.1 Novel EV subpopulations and extracellular nanoparticles

Advancements in isolation and characterisation technologies applied in EV research as well as the establishment of the MISEV guidelines have improved characterisation of EVs. This has subsequently also led to the discovery of novel (sub)populations of EVs and the discovery of extracellular nanoparticles, highlighted below.

Defensosomes are a subset of small EVs that function as a host defence mechanism in bacterial (*Staphylococcus aureus*) and viral (SARS‐CoV‐2) infection (Ching et al., 2022; Keller et al., 2020). These vesicles incorporate protein receptors on their surface during infection and serve as decoys that interfere with surface protein interactions between the host and the invading virus/bacteria (Ching et al., 2022; Keller et al., 2020). Defensosomes are thought to be around 80 nm in size, and their specific biogenesis remains unknown, however, an improved understanding of their regulation may inform clinical applications (Figure 1).

Other cell‐type specific EV populations have also been described. For example, large oncosomes (100 nm–10,000 nm in size) which are specific to cancerous cells and are formed via non‐apoptotic blebbing resulting in amoeboid movement (Di Vizio et al., 2009). Migrasomes (up to 3000 nm in size) are a trait of migrating cells, and are released from the tip of retraction fibres on cells, that are left behind as cells migrate (Ma et al., 2015). Migrasomes are described as pomegranate‐like structures, large vesicles encapsulating numerous smaller vesicles ranging 50–100 nm and their function remains to be better elucidated.

The application of asymmetric flow field‐flow fractionation has led to the discovery of a novel population of extracellular nanoparticles, called exomeres (Zhang et al., 2018). Exomeres are small (<50 nm in size) amembranous nanoparticles that are secreted by cells. Unlike all the other subtypes of EVs, exomeres are not enclosed by lipid bilayer membranes. Their cargo is distinct from EVs in the fact that they have unique proteins and biophysical properties (Anand et al., 2021). Further optimisation and investigation of exomere isolation has led to the discovery of a second distinct extracellular nanoparticle population, called supermeres (Zhang et al., 2021). Supermeres are smaller than exomeres (<25 nm) and differ in their proteomic profile to small EVs and exomeres. The biogenesis mechanisms of both exomeres and supermeres have not been elucidated.

The continuing discovery of diverse EV subpopulations and novel extracellular nanoparticles highlights the challenge that EV heterogeneity poses for proper characterisation. It also raises questions about the classification of lipid vesicles, more precisely what lipid vesicle should be classified as an ‘EV’ subtype, especially with the inclusion of diverse EVs that do not contain a lipid‐bilayer membrane. This therefore also poses the question whether LDs may be considered as a novel subtype of EV or extracellular nanoparticle.

### 2.2 Lipid droplets

The mechanisms responsible for the biogenesis and secretion of LDs is different to EVs, however, LDs, like EVs, have been shown to associate with the endoplasmic reticulum (ER) and endosomes during their biogenesis. LD biogenesis is initiated at the ER lumen via esterification of fatty acids into neutral lipids by ER localised enzymes (Olzmann & Carvalho, 2019). These neutral lipids are then placed between the bilayer of the ER resulting in lens formation. Following the development of the lens, the ER membrane protein seipin is enlisted to promote LD budding (Cartwright et al., 2015; Olzmann & Carvalho, 2019). Nascent LDs then bud off into the cytoplasm to become mature LDs. Heterogeneity in size is a common trait between both LDs and EVs (Figure 1), with the size of LDs spanning from 40 nm to 100 μm (Yang, Galea, et al., 2012). A characteristic that differs between LDs and EVs is their ability to alternate between periods of growth and depletion after being fully formed, typically in response to internal and external cues (reviewed in Olzmann & Carvalho, 2019); a capability that EVs lack. LDs are not commonly separated into different categories based on size, however, they have been given names based on where they are located within a cell, for example, nuclear lipid droplets (nLDs) that are found in the nucleus (Layerenza et al., 2013) and cytosolic lipid droplets (cLDs) that are associated with the cytoplasm (Sturley & Hussain, 2012).

LDs have been found to be secreted into the extracellular space (Lu et al., 2022), taken up by neighbouring cells (Collot et al., 2018), and implicated in influencing the tissue environment (Flaherty et al., 2019). Unfortunately, the mechanism underlying their extracellular release is unknown, however, it is plausible that LDs could take advantage of the several secretion mechanisms utilised by EVs, especially given their overlap in characteristics and their interaction with similar cellular compartments.

To date, numerous types of lipid vesicles with diverse biogenesis pathways and biophysical characteristics have been described. There is an overlap in size of EVs and LDs, as well as other similarities including their interactions with intracellular structures such as the ER and endosomes. The rise in the discovery of novel vesicles that are structurally very different to described EVs, has widened the criteria of lipid vesicles being described as EVs. As isolation technologies evolve, it is possible that the description of EVs may coevolve, and lipid vesicles that have newly discovered extracellular roles such as LDs may fall under this category.

### 2.3 Degradation of lipid vesicles

Extracellular lipid vesicles can be taken up by neighbouring cells triggering cellular responses. Once taken up by neighbouring cells, these lipid vesicles are degraded, a step that is essential to the homeostasis of cellular communication. The degradation of EVs is needed to release their cargo (proteins, lipids nucleic acids) in the recipient cell, however, the mechanisms driving this processing are largely unknown. In comparison, the degradation of LDs is well established, and is needed for the metabolic release of lipids for the cell. It is currently not known whether different degradation mechanisms are required for vesicles based on size, or vesicle composition.

Small EVs such as exosomes are hypothesised to be degraded via the autophagolysosomal pathway, with exosomes being taken up into early and late endosomes forming multivesicular bodies. Autophagosomes then fuse to the multivesicular bodies forming amphisomes, these are delivered to lysosomes leading for the eventual degradation of exosomes (You et al., 2017). Salimi et al. have reviewed the potential interactions between autophagy and EV degradation and formation, the detailed mechanisms underlying these interactions remain still unclear.

Micropinocytosis has been proposed as a means of EV degradation in cells which do not harbour antigen‐presenting abilities (Fitzner et al., 2011). This immunologically ‘silent’ manner of removing EVs in conjunction with the cell signalling ability of exosome eradication offers multiple portrayals of exosome degradation. For larger EVs such as apoptotic bodies, degradation comes in the form of efferocytosis, by tissue‐resident professional phagocytes (e.g., macrophages and immature dendritic cells) or by neighbouring non‐professional phagocytes (Yin & Heit, 2021).

Following their release and subsequent uptake into neighbouring cells, it is currently unknown if LDs are degraded, and if this is necessary for the release of cargo and ability to activate cellular pathways. However, homeostatic intracellular LDs are constantly cycling through periods of growth and depletion, with events such as starvation driving the deletion of intracellular LDs. Intracellular LDs are degraded via two mechanisms: (1) the breakdown of lipids via lipolysis and (2) a selective form of autophagy termed lipophagy (Wang, 2016; Ward et al., 2016). The most well described form of LD degradation is lipolysis, the process of breaking down lipids. During this process cytosolic lipases (including ATGL, HSL and MGL) act sequentially to catalyse the liberation of the three fatty acid moieties from TAG molecules. These free fatty acids released by this lipolytic process provide substrates for mitochondrial β‐oxidation or act as potent signalling molecules for a variety of cellular processes (Renne & Hariri, 2021), or re‐esterified back into TAG for storage when needed. LDs can also be degraded via lipophagy. This occurs when LD cargo has been tagged for degradation by protein polyubiquitination (Kirkin et al., 2009; Wang, 2016) and this tagged cargo is recognised by autophagosomal membranes via communication with the microtubule‐associated protein 1 light chain 3 (MAP1LC3) (Singh et al., 2009). While the importance and mechanism of lipolysis has been relatively well studied, mechanisms of lipophagy remain largely unknown.

The degradation of different groups of small lipid vesicles is seemingly distinct, with specific downstream effects of their degradation (i.e., release of cargo in recipient cells for EVs and fuelling metabolism for LDs). There does not seem to be any overlap in the degradation between EVs and LDs, however, it is known that LDs interact frequently with endosomes in the cytoplasm, and also share a subset of proteins involved in trafficking (Rab5c, 7, 7c and 10). It is not currently known if endosomes can take up LDs or if there is a role for LDs in delivering EVs to the endosome, particularly in providing enzymes to facilitate this process.

## 3 ISOLATION AND CHARACTERISATION OF LIPID VESICLES

Isolation of EVs and LDs with high purity and detailed characterisation is an essential step for their scientific investigation and applications. Changes in characteristics such as size, concentration, morphology and composition of vesicles may for instance be linked to disease states (Yoshikawa et al., 2019). Furthermore, a better understanding of such characteristics can guide the development of protocols and in turn lead to the standardisation of research and improve scientific rigour.

To date, only a small number of LD isolation protocols have been published (Brasaemle & Wolins, 2016; Ding et al., 2013; Mannik et al., 2014) which are all based on density gradient centrifugation. In contrast, the isolation of EVs is an active area of research and throughout the years numerous techniques have been applied (as reviewed in Gardiner et al., 2016; Liangsupree et al., 2021). To promote standardisation of EV isolation protocols and reporting of protocols and results, the MISEV guidelines were introduced (Théry et al., 2018).

Given the similarities between EVs and LDs, techniques used for EV isolation could be adapted and integrated for LD isolation and vice versa. In this section, we will provide a concise overview of the predominant techniques used for isolation and characterisation of EVs and LDs.

### 3.1 Isolation of EVs

A wide variety of methods have been described to isolate EVs. Primarily, EVs are isolated from cell culture media or biofluids, and isolation focuses on removing extracellular contaminants such as protein, cell debris and other extracellular nanoparticles (e.g., lipoproteins). EV isolation techniques commonly rely on differences in biophysical characteristics of EVs and the contaminants.

Differential ultracentrifugation was the first method used to isolate EVs and relies on multiple centrifugation steps of increasing force to remove cells, proteins, and other debris before a high‐speed spin to pellet EVs. Alternative methods, such as size exclusion chromatography (SEC), rely on particle size, while density gradients (e.g., sucrose and Optiprep) rely on separating particles from contaminants by their density (Phillips et al., 2021). The choice of EV isolation method is often made on the basis of desired purity and yield, which can be influenced by the intended downstream applications (Lai et al., 2022). The isolation of ‘pure’ EVs has been difficult to achieve, especially due to the presence of contaminants with overlapping biophysical characteristics, such as lipoproteins and complexity arising from the presence of EV subpopulations (Willms et al., 2016) with similar biophysical characteristics but different composition.

One approach to this issue is the development of isolation methods based on composition that can target either EVs or other extracellular nanoparticles (Phillips et al., 2021). Affinity capture of EVs has been employed with methods utilising antibody‐conjugated magnetic beads or affinity chromatography to isolate EVs based on membrane‐bound proteins, such as membrane‐bound tetraspanins (Liangsupree et al., 2021). Importantly, separating EVs by their composition allows for isolation of cell‐type‐specific EVs from complex mixtures, such as blood or tissue, containing EVs originating from various cell types throughout the organism. Cell type‐specific markers could be a useful target to isolate cell‐specific EVs. Examples of this include the use NCAM or L1CAM for the isolation of neuronal‐specific EV, however, these markers are controversial due to them not solely being found in the brain, demonstrating the complexity of isolating cell‐specific EVs from biofluid (Gomes & Witwer, 2022). Alternative less specific composition‐based isolation methods through an affinity ligand such as heparin may also be employed (Balaj et al., 2015).

Due to the wide variety of EV isolation techniques available, the possibility of combining multiple techniques targeting different EV characteristics has been explored. One such example utilised SEC with ion exchange chromatography (IEX) to separate EVs from lipoprotein populations in human serum based on differences in size and charge (Van Deun et al., 2020).

Currently, density gradient ultracentrifugation is arguably the most widely used method for balancing EV purity and yield. This method is largely successful in separating EVs from more dense particles such as protein aggregates, however, it results in low specificity as it does not allow for separation of EV populations (Cocozza et al., 2020). Density gradient isolation of EVs does not allow for complete removal of lower density vesicles such as lipoproteins which are similar in both size and density to EVs. It is therefore likely that density gradients are not suitable for the separation of LDs and EVs. The density of LDs is very low (less than 1 g/mL) as a result of their high neutral lipid content, comparatively lipoproteins can range from less than 0.95 g/mL to 1.21 g/mL which both overlap with the density of EVs (1.06–1.21 g/mL) (Brasaemle & Wolins, 2016; Brennan et al., 2020; Onódi et al., 2018).

#### 3.1.1 Isolation of tissue derived EVs

In addition to the isolation of EVs from cell culture supernatant and biofluids, there has been a growing interest in isolating EVs directly from tissue, as they may provide insight into tissue‐specific disease pathology such as cancer, ALS and Alzheimer's disease (Crescitelli et al., 2020; Huang et al., 2022; Perez‐Gonzalez et al., 2012; Su et al., 2021; Vassileff et al., 2020; Vella et al., 2017). Isolation of EVs from tissue is difficult as EVs exist within the interstitial space between cells, which is comprised of a protein matrix that ‘trap’ EVs. Disruption of this matrix needs to be gentle as damage to cells may release intracellular components and may contribute to additional contamination (Crescitelli et al., 2021). Most tissue‐derived EV protocols rely on an enzyme treatment, typically collagenase, to degrade the matrix and ‘release’ the EVs. Following treatment, the sample undergoes a low‐speed centrifugation spin or a filtration step that separates cells and debris. Finally, an EV isolation protocol is performed, most commonly based on density gradient ultracentrifugation, although SEC, differential ultracentrifugation, and ultrafiltration have been utilised in this context (Huang et al., 2020). As the isolation of EVs from tissue relies on methods that may result in the lysis of cells, intracellular lipid vesicles such as LDs may be co‐isolated.

### 3.2 Isolation of LDs

Compared to the isolation of EVs, LD isolation techniques are not well described, especially in mammalian cells, with centrifugation being the only described method of isolating LDs (Brasaemle & Wolins, 2016; Ding et al., 2013). One major difference between EV and LD isolation is the starting material used for isolation, LDs are typically isolated from cell or tissue lysates and not from cell culture media or biofluids.

Density gradient centrifugation is the main technique used for LD isolation. LDs can be recovered from the low density fractions, however, this technique has been shown to preferentially isolate larger LDs (which are the very top fraction), as a result smaller LDs can be lost (Ding et al., 2013; Mannik et al., 2014). Additionally, low density membrane structures (e.g., ER, mitochondria, and endosomes) can be co‐isolated, further increasing the difficulty of isolating pure LDs (Ding et al., 2013). Larger LDs may also be damaged during cell homogenisation steps (Ding et al., 2013). With the increased interest in LDs, there is a need for new techniques that allow for the isolation of LDs with high purity. Unlike the stringent guidelines for EV isolation, there are currently no guidelines in place for the isolation of LDs. Methods of isolating LDs overlap with methods used to isolate EVs, particularly when isolating based on density. It is possible that there is co‐isolation of these two lipid vesicles however, to our knowledge screening on LD contaminants in EV preps and vice versa is not commonly performed.

## 4 PHYSICAL CHARACTERISATION OF LIPID VESICLES

Following the isolation of lipid vesicles, numerous techniques can be used for their characterisation. Information on characteristics such as size, concentration, morphology and composition can provide valuable insights. Characterisation of EVs has played a key role in EV research and as a result, multiple techniques have been developed and optimised for application in EV research. Interestingly, most of the methods developed in the EV field have not been applied in LD research and LD researchers have developed alternative methods that have not been applied in EV research (as reviewed by Martins et al., 2018). Given the similarities between EVs and LDs there is clear potential to adopt the methods developed in the EV and LD fields and in this way improve characterisation of both classes of lipid vesicles.

In contrast to the EV field that has various techniques to quantify isolated EVs (e.g., nanoparticle tracking analysis (NTA) and tunable resistive pulse sensing (TRPS), described below), the LD field mainly performs analysis of LDs present within in the cell (i.e., not on isolated LDs). Microscopy is commonly applied in the LD field with many protocols employing fluorescent dyes that stain the neutral lipid content in LDs. Fluorescent neutral lipid dyes such as Bodipy (493/503) (Spangenburg et al., 2011), Nile Red (Cohen et al., 2017), LD540 (Spandl et al., 2009), LipidTox (Wilson et al., 2021) and Autodot (Hariri et al., 2018) are frequently used to label LDs in live and fixed cells. These dyes are diffusible, and are easily able to cross cellular membranes, but only fluoresce in a neutral lipid environment. Bodipy (493/503) is the most commonly used LD dye and has been used in many studies including flow cytometry and microscopy, and is also used as a method of sizing LDs (Cohen et al., 2017; Monson, Crosse, et al., 2021). Unfortunately, due to these lipid dyes being diffusible, they cannot be used in some instances, therefore fluorescently labelled antibodies targeting LD protein markers such as ADRP are often used (Wilson et al., 2021). Microscopy‐based approaches which are not based on fluorescence, such as electron and atomic force microscopy (AFM), are also used.

Additionally, stimulated Raman light scattering microscopy has been used to quantify LDs at the single cell level (Cao et al., 2016). This method does not use labelling therefore overcomes the limitations arising from using lipophilic dyes (Cao et al., 2016). Other microscopy types such as AFM can be used to characterise LDs, by using a scanning probe on the surface of the LD. For this technique, pure isolated LDs are placed in suspension in order to maintain their structure allowing for quantitative analysis of their height and diameter (Carvalho et al., 2012).

### 4.1 Nanoparticle tracking analysis (NTA)

Nanoparticle tracking analysis (NTA) is a light‐scattering technique commonly used for the sizing and enumeration of EVs (Gardiner et al., 2013). The diffusion of vesicles in suspension as a result of their Brownian motion is recorded over time and used for the calculation of their hydrodynamic size. Recent improvements to the NTA technology enable the incorporation of immunolabelling and the detection of EV surface epitopes (Dragovic et al., 2011; Thane et al., 2019). To our knowledge Muratore and colleagues published the only manuscript describing the measurement of LDs with NTA, describing NTA analysis on LDs isolated from mouse liver tissue and concluding that the technique represents an improvement over time‐consuming and low throughout imaging‐based size measurements of LDs (Muratore et al., 2018).

### 4.2 Tunable resistive pulse sensing (TRPS)

TRPS is a particle sizing method that detects changes in current as individual particles pass through a nanopore. TRPS can measure particle size, concentration and zeta potential (Vogel et al., 2016). As TRPS measures individual particles, it is a highly accurate sizing technique. However, TRPS utilises nanopores that require time‐consuming optimisation and calibration to determine the necessary pore size and avoid clogging of the nanopore (Akers et al., 2016). TRPS has been employed as an EV sizing and concentration tool; however, no studies have used TRPS to measure LDs to date.

### 4.3 Flow cytometry

Flow cytometry has been more widely used in the EV field in recent years, especially for the characterisation of large EVs such as apoptotic bodies (Atkin‐Smith et al., 2015; Caruso & Poon, 2018; Serrano‐Heras et al., 2020). However, size has been a limiting factor of this characterisation method for smaller EVs as most flow cytometers are suitable for analysis in the μm not nm range. Newer more sensitive instruments have been developed that focus on vesicles in the nm range, making flow a more applicable method for the study of small EVs (Arab et al., 2021). A combination of capture antibody beads with fluorescently labelled detection antibodies can also be used to circumvent the size limitation of conventional flow cytometry for EV analysis, and this combination allows for the analysis of proteins expressed on the surface of EVs. Recently, several groups described novel strategies to stain purified EVs for flow cytometry (Morales‐Kastresana et al., 2017), optimised imaging flow cytometry for analysis of EV samples (Görgens et al., 2019), developed new methodologies for the detection and sorting of EVs (Morales‐Kastresana et al., 2019). To date, flow cytometry has not been utilised for the characterisation of isolated LDs, however, given the success of this technique in the EV field it is highly likely this method would be transferable to LD characterisation.

### 4.4 Microscopy‐based investigations (TEM, Cryo‐TEM and AFM)

Transmission electron microscopy (TEM) is a vital tool for the study of EV morphology (Rupert et al., 2017). TEM has also been used for the characterisation of LDs; however, the sample preparation typically used for this analysis involves fixation and staining the sample, resulting in dehydration of the vesicles and does not preserve the native morphology of isolated LDs and EVs (Mahamid et al., 2019). Alternativity, cryogenic transmission electron microscopy (cryo‐TEM) is a powerful tool for studying native morphologies. Cryo‐TEM sample preparation fixes samples by plunging the grids into liquid ethane and embedding the sample in vitreous ice. This rapid freezing step prevents the formation of disruptive ice crystals, so sample integrity and morphology are preserved. Cryo‐TEM is extensively used in EV research and has allowed researchers to detect EV subpopulations with diverse morphologies (Coleman et al., 2012; Zabeo et al., 2017).

Unfortunately, the membrane monolayer of LDs makes the visualisation of LDs under cryo‐TEM challenging, but it is possible to observe both LDs present in cells and isolated LDs (Mahamid et al., 2019; Tauchi‐Sato et al., 2002). To enhance TEM, immunogold labelling of protein markers on the surface of vesicles can make it easier to detect vesicles and distinguish populations. Immunogold TEM has a long history of being used to study EVs (Harding et al., 1983) and has been used to investigate the localisation of protein markers on the surface of LDs (Robenek et al., 2005). Cryo‐TEM imaging is a powerful technique; however, it is not widely employed due to its time‐consuming nature, required expertise for imaging and analysis, and the relatively small number of vesicles that can be observed in images.

AFM is a widely available and accessible alternative that can obtain label‐free, 3D, quantitative information of EVs at sub‐nanometer scale resolution (Sharma et al., 2010). AFM is often used to characterise EVs, and recently the implementation of Raman spectroscopy probes has allowed for the additional evaluation of EV composition (e.g., chemical properties and chemical dynamics) (Kim et al., 2019). These microscopy‐based characterisation techniques could collectively provide valuable information about novel populations of LDs that are not currently classified.

Based on the aforementioned, there is a clear opportunity to integrate the diverse isolation and characterisation techniques applied and developed in the EV and LD fields. This integration will benefit both fundamental and applied EV and LD research and allow researchers to address challenges such as the heterogeneity of lipid vesicles.

## 5 EV AND LD HETEROGENEITY

As highlighted in Figure 1, cells form and release different populations of lipid vesicles. In their 1987 publication describing EV formation during reticulocyte maturation, Johnstone et al. already raised the question whether each individual vesicle released by a cell contains a mixture of all the externalised components, or, if instead a mixed population of vesicles is released (Johnstone et al., 1987). In other words, is each individual EV of identical composition, or are EVs with diverse composition and biological function released. This question has been at the core of a large amount of EV research throughout the years and it has become apparent that EVs are a highly heterogenous population of lipid vesicles. Even within previously assumed homogenous EV populations, distinct subpopulations of EVs with varying composition and biological effects have been discovered (Willms et al., 2016). The investigation of EV populations has been hampered by the fact that isolation is performed based on biophysical characteristics, whereas the theoretical classification of EV populations is based on biogenesis. Furthermore, only a small number of proteins has so far been shown to be applicable to distinguish between unique EV populations (Kowal et al., 2016).

Heterogeneity in size and composition has also been of interest to the study of LDs. This interest has been sparked by findings that indicate distinct functions between LDs of different sizes (Zhang et al., 2016). Interestingly, although LDs have one well described mechanism of biogenesis, activators of this biogenesis pathway can differ and are thought to influence LD protein composition (Bosch et al., 2020; Liu et al., 2019).

A better understanding of EV and LD heterogeneity is needed to more precisely establish the physiological roles of these lipid vesicles and improve their potential application as therapeutics and diagnostics (Tkach et al., 2018). Advances in isolation and characterisation techniques, such as single‐vesicle analysis and imaging approaches, will benefit studies on the composition of EVs and LDs and allow researchers to establish markers that can be used to differentiate between unique EV and LD populations (Bordanaba‐Florit et al., 2021; Willms et al., 2018).

## 6 THE COMPOSITION OF LIPID VESICLES

### 6.1 General cargo of EVs and lipid droplets

Advancements in the isolation of lipid vesicles have progressed the current understanding of their cargo, including their proteome, lipidome, metabolome and a detailed understanding of the nucleic acids they carry; sequentially increasing knowledge of their roles in cellular communication. The localisation of the cargo associated with lipid vesicles is important for their role in cell‐to‐cell communication, with cargo either being internalised within the phospholipid membrane, or localised on the outside of the vesicle, potentially associated with the membrane. Vesicles that contain cargo on the outside, such as proteins and receptors, have the ability to activate recipient cells without being degraded to release cargo. On the other hand, internalised cargo is protected from the extracellular environment, and for cargoes such as RNA which are easily degraded, this is particularly important. Internalised cargo is released when the phospholipid membrane is broken down, usually occurring following uptake of the vesicle by a recipient cell.

**FIGURE 2:**
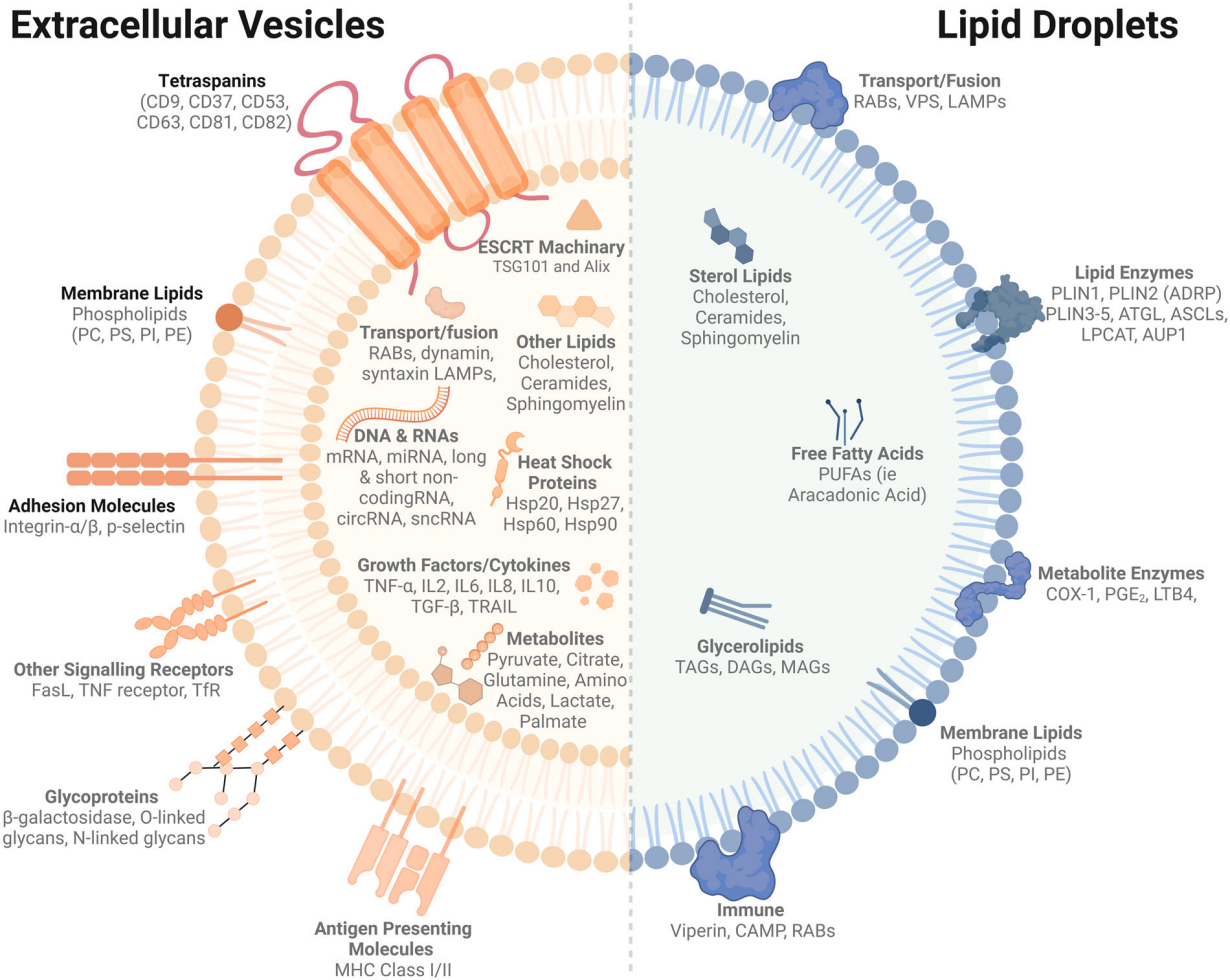
General cargo of EVs and LDs. Schematic diagram of the cargo that has been described in EVs and LDs. EVs contain a range of proteins, protein and signalling receptors, lipids, nucleic acids and metabolites, however, the less studied LDs have only been shown to contain proteins and lipids. The localisation of the cargo also differs, with proteins being internalised inside EVs compared to being exclusively localised to the membrane of LDs. cAMP,  3′,5′‐cyclic adenosine 5′‐monophosphate; LAMP, lysosome‐associated membrane glycoproteins; PC,  phosphatidylcholine; PE, phosphatidylethanolamine; PI, phosphatidylinositol; PS, phosphatidylserine; TfR, transferrin receptor; TNF, tumour necrosis factor.

The cargo of lipid vesicles can differ substantially between groups. For example, EVs carry a very wide variety of cargo, including nucleic acids, proteins, receptors, lipids, enzymes, metabolites and toxins enclosed within a phospholipid bilayer membrane (Harischandra et al., 2017; Maas et al., 2017) (Figure 2). LDs on the other hand have only been described to carry internal neutral lipids, enclosed by a phospholipid monolayer decorated with external proteins (Figure 2) (Kory et al., 2016). To date, the cargo that is most commonly used and recommended by the MISEV guidelines to use as purity markers are proteins, and include cytosolic accessory proteins such as Alix, VPS4A/B and Flotillins‐1 and 2, tetraspanins such as CD9, CD81 and CD63 and the Heat shock proteins HSC70 (HSPA8), and HSP84 (HSP90AB1). Nucleic acids such as DNA and RNA are also commonly described in EV populations, however, no specific nucleic acid markers have been associated with EV subtypes (Théry et al., 2018).

Unlike the well‐studied cargo of EVs, there has been little research into the cargo of LDs; additionally, most LD‐omics studies are performed in yeast models which are a limitation in this field (Athenstaedt et al., 1999, 2006; Grillitsch et al., 2011). Unfortunately, to date there has been no examination of the nucleic content of LDs, which is a gap in knowledge of the cargo LDs carry (Figure 2). LDs, however, have a complex complement of proteins which endow them with their many functions, mainly involved in transport, lipid synthesis and more recently disease (Figure 2). The most common LD resident proteins are the perilipin (PLIN) family which are mainly involved in lipolysis and cellular lipid homeostasis. LD protein compositions have some cell and tissue specificity (Zhang & Liu, 2019), however, this could also be due to LD isolation techniques not being consistent in the field. Unfortunately, to our knowledge, no studies to date have examined other cargo of LDs such as nucleic acids, which might give insights into their functions within cells and their role in extracellular communication.

### 6.2 The extensive proteome overlap of EVs and LDs

A major driver in EV and LD function is their proteome. Both EVs and LDs have vast proteomes which can differ from the cell and tissue types they originate from. There are known protein overlaps of lipid vesicles, including members from the RAB and LAMP families that localise to both EVs, LDs and other lipid vesicles such as endosomes (Bottger et al., 1996; Langemeyer et al., 2020). In fact, some RAB proteins are regulators of LD formation and drive communication with other organelles (Li & Yu, 2016) similar to their function in EVs, which involves vesicular trafficking and their biogenesis (Blanc & Vidal, 2018).

Over the past 10 years, the use of mass‐spectrometry based proteomics technologies has enabled in‐depth proteome profiling of EVs in particular, leading to the discovery of an increasing number of EV‐associated proteins and potential biomarkers of disease. This has sparked interest and extensive research into the EV proteome which has allowed for the establishment of the proteome database, Vesiclepedia, showcasing almost 1000 mammalian proteomic screens that have identified unique EV‐associated proteins across many models. This resource has identified recurring proteins such as Alix and TSG101 (identified 399 and 255 times respectively) which are now commonly used as small EV purity markers (Théry et al., 2018; Willms et al., 2018). In comparison, the LD proteome is not well described, with only 14 published mammalian LD proteomes to date. EVs share proteins with other lipid vesicles such as lipoproteins (Simonsen, 2017), however, no comparison studies have looked at the overlap of EVs and LDs, despite there being a number of proteins that they are known to share.

As described earlier, the ESCRT pathway is one known pathway of EV biogenesis. Alix (PDCD6IP) and TSG101 are key players hypothesised to be involved in the budding and abscission process occurring in this pathway. Alix is the number one protein identified on EVs according to the Vesiclepedia database (Kalra et al., 2012), and is widely accepted as a purity marker of small EVs due to its role of sorting cargo into exosomes (Raiborg & Stenmark, 2009). Interestingly, Alix and other EV markers have also been identified in multiple published LD proteome databases (Bersuker et al., 2018; Bosch et al., 2020; Rösch et al., 2016), (Table 1). The function of Alix in LDs is unknown, however, in a very recent publication examining the proteome of LDs, Alix was identified, and was clustered with LD resident proteins (PLIN3, AUP1 and RAB family members) (Bosch et al., 2020) via high‐correlation protein clustering analysis. As LDs have newly been described to be secreted from cells, it is possible that Alix might play a role in the biogenesis and/or the secretion of LDs, and they could potentially be utilising the ESCRT pathway for secretion.

**TABLE 1:** Comparison of published LD proteomes to top 50 EV proteins

Protein	Model	Function
Proteins that are present in EVs and LDs		
**PDCD6IP** **(Alix)** *	***EV***: *(Mouse, rat, human)*	Involved in Exosome biogenesis (Baietti et al., 2012) Used as exosome marker (Duijvesz et al., 2013)
	***LD***: *(human* (Bersuker & Olzmann, 2017)*, mouse* (Bosch et al., 2020; Wang et al., 2015)	unknown
**AnxA2** *	***EV***: *(mouse, human, rat)*	Recruits miRNAs to EVs (Hagiwara et al., 2015) Involved in exogenous RNA binding in serum EV (Tapparo et al., 2021)
	***LD***: *(mouse* (Bersuker & Olzmann, 2017; Huina Zhang et al., 2011)*, human* (Bersuker & Olzmann, 2017; Bouchoux et al., 2011))	unknown
**AnxA6**	***EV*** *(mouse, human, rat)*	Scaffolding protein on EVs
	***LD*** *: mouse* (Wang et al., 2015)*, human* (Bersuker & Olzmann, 2017)	Overexpression results in elevated LD size and numbers (Cairns et al., 2017)
**EEF2**	*EV (mouse, rat, human)*	Unknown
	*LD: mouse* (Wang et al., 2015)*, human* (Saka et al., 2015); (Bersuker & Olzmann, 2017)	Unknown
**TSG101**	*EV: (Mouse, human, rat, horse)*	EV purity marker (Théry et al., 2018) EV secretion compromised when TSG101 is inhibited (Colombo et al., 2013)
	*LD: mouse* (Bosch et al., 2020)	Transfer of fatty acids from LDs (Wang et al., 2021)
**Hspa8** *	*EV: (rat, mouse, human)*	Involved in the breakdown of clathrin from vesicles (Stricher et al., 2013)
	*LD: Human* (Bersuker & Olzmann, 2017; Bouchoux et al., 2011; Rösch et al., 2016)	Involved in PLIN2 (ADRP) phosphorylation (Kaushik & Cuervo, 2016)
**RAP1B**	*EV: (mouse, human, rat, salmon, sheep)*	Unknown
	*LD: Mouse* (Wang et al., 2015)*, Human* (Bersuker & Olzmann, 2017; Bouchoux et al., 2011; Saka et al., 2015)	Unknown
**ATP1a1**	*EV: (mouse, human, rat, drosophila)*	Unknown
	*LD: Mouse* (Wang et al., 2015)*, Human* (Bersuker & Olzmann, 2017; Menon et al., 2019)	Unknown
**CCT2**	*EV: (mouse, human, rat)*	It has been reported that CCT2 from umbilical cord mesenchymal stem cells EVs influences CD154 expression (Zheng et al., 2020)
	*LD: Mouse* (Wang et al., 2015)*, Human* (Bersuker & Olzmann, 2017; Bouchoux et al., 2011)	Unknown
**ALDOA**	*EV (mouse, rat, human*	Promotion of glycolytic processes (Wang et al., 2020)
	*LD: Mouse* (Wang et al., 2015; Zhang et al., 2011)*, Human* (Bersuker & Olzmann, 2017; Rösch et al., 2016)	unknown
**CLTC**	*EV (mouse, human, rat*	Involved in exosome uptake in macrophages (Wan et al., 2020)
	*LD: Mouse* (Wang et al., 2015)*, Human* (Bersuker & Olzmann, 2017)	unknown
**GNB2**	*EV (mouse, human, rat)*	Unknown
	*LD: Mouse* (Wang et al., 2015) *Human* (Bersuker & Olzmann, 2017)	Unknown
**MYH9**	*EV (mouse, human, rat)*	Interferes with exosome release (Lv et al., 2020)
	*LD*: *Mouse* (Liu et al., 2019; Wang et al., 2015) *Human* (Bersuker & Olzmann, 2017)	Depletion of MYH9 increases LD size and compromises lipid breakdown and fewer LD clusters (Pfisterer et al., 2017)
**SLC3A2**	*EV (mouse, human, rat)*	unknown
	*LD* *Mouse* (Wang et al., 2015) *Human* (Bersuker & Olzmann, 2017; Saka et al., 2015)	unknown
**LDHA**	*EV (mouse, human, rat, salmon)*	Unknown
	*LD*: *Mouse* (Wang et al., 2015) *Human* (Bersuker & Olzmann, 2017; Bouchoux et al., 2011; Dahlhoff et al., 2015)	Induces LD growth (Goo et al., 2017)
**RAB5C**	*EV (mouse, human)*	Integral for cargo sequestration (Stenmark, 2009)
	*LD: (Mouse* (Wang et al., 2015)*, Human* (Bersuker & Olzmann, 2017))	Rab5c is an isoform of Rab5 which localises to LDs and recruits EEA1(P. Liu et al., 2007)
Proteins specific to EVs		
**CD81**	(Kalra et al., 2012)	positive and negative regulator of homotypic or heterotypic cell‐cell fusion processes (Andreu & Yáñez‐Mó, 2014).
**CD63**	(Kalra et al., 2012)	Involved in ESCRT dependent and independent cargo loading into late endosomes (van Niel et al., 2011).
**CD9**	(Kalra et al., 2012)	Involved in EV uptake in cancer‐associated fibroblasts (Nigri et al., 2022).
**SDCBP** **(Syntenin)**	(Kalra et al., 2012)	Involved in the biogenesis of exosomes (Baietti et al., 2012).
**ACTB**	(Kalra et al., 2012)	EV specific function unknown
Proteins specific to LDs		
**ADRP (PLIN2)**	(Imamura et al., 2002; Yang, Ding, et al., 2012)	Assists in the storage of neutral lipids within the LDs (Imamura et al., 2002)
**PLIN1**	(Itabe et al., 2017; Yang, Ding, et al., 2012)	Lipid metabolism (Hansen et al., 2017)
**AUP1**	(Spandl et al., 2011)	Regulates lipid metabolism and induces lipid accumulation (Chen et al., 2022)
**BSCL2** **(Seipin)**	(Salo et al., 2016)	Plays a crucial role in the formation of LDs, particularly budding from ER (Kim et al., 2022).

* Proteins appear in the top 10 EV proteins on Vesiclepedia (Kalra et al., 2012).

The ESCRT pathway member, TSG101 has also been identified in multiple LD proteomes (Bersuker et al., 2018; Bosch et al., 2020) (Table 1). Unlike Alix, TSG101 has been suggested in the past to have interactions with LDs, where TSG101 promotes fatty acid trafficking from LDs to the mitochondria (Wang et al., 2021). TSG101 underpins MVB formation, critical for EV secretion which is ATP dependent (Tran et al., 2009), and it is plausible that TSG101 may be promoting ATP synthesis via LD‐Mitochondria interactions driving this process. Other proteins shown to overlap between EVs and LDs in published databases include multiple proteins involved in biogenesis (Alix, AnxA6), cargo recruitment (AnxA2, AnxA6), transport (TSG101, EEF2, CLTC) and also several with unknown functions (RAP1B, SLC3A2, GNB2, EEF2) (Table 1).

In order to gain a more detailed view of the percentage overlap between the proteome of EVs and LDs we also performed a comparison of the seven published LD proteomes against the top 100 EV proteins on the Vesiclepedia database (Table 1). Interestingly, there is a large protein overlap observed between the LD proteome database containing the most proteins (U2OS cells), and the top 100 EV proteins (75% overlap). It is clear that the correlation decreased as the size of the LD proteomic databases decreased, indicating that there is likely a significant overlap between both LD proteomes and those of EVs (Table 1).

From this comparison, there were also a handful of proteins unique to EVs (i.e., not present in any published LD proteomes), mainly comprised of tetraspanins (CD81, CD63, CD9) and Syntenin. These proteins are currently suggested as EV markers by the MISEV guidelines (Théry et al., 2018), and their absent in LD proteomes suggests they would make good markers of EV purity (Table 1). There were also LD specific proteins identified, such as members from the PLIN family; PLIN1, PLIN2 also known as ADRP, AUP1 and seipin were also unique to LD proteomes, and are not found in the Vesiclepedia database (Table 1). Due to the extensive protein overlap of these two lipid vesicles, it is important to identify unique proteins to each group as this will be vital in their downstream characterisation.

The isolation of EVs from tissue samples is more complex than from cell culture models as it involves homogenisation of tissues, thus making it difficult to confirm that isolated EVs are pure, and solely derived from the extracellular space (Théry et al., 2018). As such, the MISEV guidelines have acknowledged the difficulty in isolation of pure EVs from tissue samples which is a technological aspect that requires optimisation. Of concern, the isolation of EVs from tissue samples could cause cell breakage via mechanical disruptions and could therefore increase the possibly of co‐isolating other lipid vesicles such as LDs (Théry et al., 2018). We, therefore, recommend the inclusion of a more diverse range of purity markers, including markers of LDs.

As seen in Table 2, there is a more considerable overlap (up to 96%) of LD proteomes to EVs isolated from tissue samples (Crescitelli et al., 2020) (Table 2). Of these, the greatest overlap (96.84%) was observed in the LD proteome of cells infected with Hepatitis C virus (HCV) (Rösch et al., 2016) compared to EVs isolated from human metastatic melanoma (Crescitelli et al., 2020) (Table 2). We found this particularly interesting for two reasons: firstly, LDs are commonly increased in cancerous cells, and accumulate during virus infection (Antunes et al., 2022; Monson, Crosse, et al., 2021; Monson, Trenerry, et al., 2021). This high abundance of LDs in tissue samples increase the difficultly of successful isolation of pure EVs particularly in small, low density EV preps. The presence of the main LD resident protein PLIN2 (ADRP), as well as other PLIN family members was observed in both pure tissue‐derived EV fractions, which further raises the question whether there is co‐isolation of LDs and EVs occurring. Secondly, it is also possible that this extensive overlap is attributed to the contamination of the EV fraction with other lipid vesicles, namely lipoproteins, which pose a significant difficulty in the isolation of EVs (Brennan et al., 2020; Simonsen, 2017). A significant increase of ApoE (a component of lipoproteins) was observed in small low density EVs and small EV preps and was also observed in the LD fractions of HCV infected Huh‐7 cells (Rösch et al., 2016). It is possible that the additional overlap observed between these two datasets is likely a result of lipoprotein and other lipid vesicle contamination.

**TABLE 2:** Comparison of mammalian LD proteomes to a tissue derived EV proteome

			Tissue derived EVs	
Mammalian LD proteomes	Total # proteins	% Similarity to top 100 EV proteins	Human metastatic melanoma tissue (Crescitelli et al., 2020)	Human ALS Motor Cortex (Vassileff et al., 2020)
**U2OS** (Bersuker et al., 2018)	1302	75%	1138 **(87.40%)**	492 **(37.8%)**
**Huh‐7** (Bersuker et al., 2018)	699	43%	633 **(90.5%)**	299 **(42.77%)**
**Huh‐7** *[+ HCV]* (Rösch et al., 2016)	316	48%	306 **(96.84%)**	205 **(64.87%)**
**HeLa** *[+ Chlamydia trachomatis]* (Saka et al., 2015)	88	16%	78 **(88.6%)**	49 **(55.7%)**
**Caco‐2/TC7 enterocytes** (Bouchoux et al., 2011)	98	17%	87 **(88.78%)**	54 **(55.1%)**
**Sebocytes** (Dahlhoff et al., 2015)	1586	59%	1423 **(89.72%)**	524 **(33.03%)**
**Macrophage** *[+Mycobacterium tuberculosis]* (Menon et al., 2019)	437	50%	384 **(87.9%)**	288 **(37.9%)**

Clearly this is an underexplored area of research, and further work needs to be performed to fully elucidate whether the protein crossover between EVs and LDs is due to contamination of isolated fractions, LDs interacting with EV secretion, or if the proteome of these two lipid vesicles is more similar than previously thought. However, the possible localisation of Alix and TSG101 to LDs makes the isolation of pure EVs extremely difficult using current methods, given EVs and LDs are also similar in size, and size and cargo are the only two methods used to isolate and validate pure EV populations. It is clear that the overlap between the proteomes of EVs and LDs is quite extensive. Most of the overlapping proteins between the two particles play roles in the ESCRT pathway, and although the mechanism of LD secretion is currently unknown, this may suggest similar secretion pathways for both EVs and LDs. However, we cannot rule out the possibility that this overlap in proteomes could be due to suboptimal isolation techniques applied in both fields, especially those that are isolating based on size, and validating based on the presence of Alix and TSG101. Indeed, although there are unique LD proteins, as identified in Table 1, these LD proteins (PLIN family members) were sporadically detected in the proteomes of tissue derived EVs, further supporting the possibility of LDs contaminating EV isolates.

## 7 CONCLUSIONS AND PERSPECTIVES

Lipid vesicles play important roles in biology and recent improvements in technologies have led to an increased and more detailed description of their characteristics, and a better understanding of their roles in cellular communication. To date, most research has focussed on exploring individual classes of lipid vesicles and little interdisciplinary research of lipid vesicles of different types has been conducted. As outlined in this review, similarities between two lipid vesicle populations; EVs and LDs, highlight the potential synergy between these research fields.

The recent findings demonstrating the release of LDs within small EVs (AdExos) from adipocyte tissue (Flaherty et al., 2019) as well as reports of LDs being secreted from cells in milk ducts (Lu et al., 2022) have prompted the question of the involvement of LDs in intercellular communication, a biological function though to be predominantly carried out by EVs. Interestingly, multilamellar vesicles have been observed in purified samples of EVs, suggesting that packaging of vesicles within vesicles can occur (Coleman et al., 2012; Emelyanov et al., 2020; Höög & Lötvall, 2015; Yuana et al., 2013; Zabeo et al., 2017).

There has been increasing interest in the application of lipid vesicles for drug delivery, including delivery of nucleic acids for gene therapy, and novel vaccines (Cecchin et al., 2023; Santos & Almeida, 2021). EVs have been harnessed as novel drug delivery vehicles and various engineering strategies have been used to optimise their characteristics for this application (Armstrong et al., 2017). The potential novel application of LDs as a drug delivery vehicle has prompted interest in engineering LDs, and recently, methods have been optimised to make artificial LDs which could be harnessed for this application (Ma et al., 2021). Fully understanding the properties of LDs, especially mechanisms that allow them to move between cells is vital for this research to progress.

It has already been reported that LDs can play a role in intercellular communication via transfer through tunnelling nanotubes formed between cells (Astanina et al., 2015). Furthermore, a recent preprint by Genard G. & Tirinato et al. describe a correlation between LD and EV biogenesis (Genard et al., 2022). In this review, we additionally highlight the overlap between the proteomes of EVs and LDs and report that proteins such as Alix and TSG101, which are typically viewed as EV specific proteins, can be detected in LD isolates. Further research into the exact localisation of these proteins is required for understanding the interaction between these two lipid vesicles and can help determine the roles both vesicles play in various physiological and pathophysiological processes. Taken together, these findings indicate an unexplored overlap and potential interaction between EV and LDs. As outlined throughout the review, the integration of knowledge and techniques used in both fields can support the exploration of this interesting interaction and shed light on the potential novel role of LDs in intercellular communication.

## CONFLICT OF INTEREST STATEMENT

The authors declare no conflicts of interest.
